# Gray Matter Changes in Parkinson’s and Alzheimer’s Disease and Relation to Cognition

**DOI:** 10.1007/s11910-019-1006-z

**Published:** 2019-11-13

**Authors:** Lenka Krajcovicova, Patricia Klobusiakova, Irena Rektorova

**Affiliations:** 1 Applied Neuroscience Research Group, CEITEC, Masaryk University, Kamenice 5, Brno, Czech Republic; 2 First Department of Neurology, Faculty of Medicine, St. Anne’s University Hospital, Masaryk University, Pekarska 53, Brno, Czech Republic; 3 Faculty of Medicine, Masaryk University, Brno, Czech Republic

**Keywords:** Parkinson’s disease, Alzheimer’s disease, Gray matter atrophy, Cognition, Structural magnetic resonance imaging

## Abstract

**Purpose of Review:**

We summarize structural (s)MRI findings of gray matter (GM) atrophy related to cognitive impairment in Alzheimer’s disease (AD) and Parkinson’s disease (PD) in light of new analytical approaches and recent longitudinal studies results.

**Recent Findings:**

The hippocampus-to-cortex ratio seems to be the best sMRI biomarker to discriminate between various AD subtypes, following the spatial distribution of tau pathology, and predict rate of cognitive decline. PD is clinically far more variable than AD, with heterogeneous underlying brain pathology. Novel multivariate approaches have been used to describe patterns of early subcortical and cortical changes that relate to more malignant courses of PD.

**Summary:**

New emerging analytical approaches that combine structural MRI data with clinical and other biomarker outcomes hold promise for detecting specific GM changes in the early stages of PD and preclinical AD that may predict mild cognitive impairment and dementia conversion.

**Electronic supplementary material:**

The online version of this article (10.1007/s11910-019-1006-z) contains supplementary material, which is available to authorized users.

## Introduction

Parkinson’s disease (PD) and Alzheimer’s disease (AD) are the two most common neurodegenerative diseases. As compared to AD, PD is clinically far more variable with heterogeneous underlying brain pathology [1]. In clinical practice, the diagnosis of PD and AD is based on the presence of typical clinical symptoms (see established clinical criteria, [1, 2]). Recently, the use of biomarkers has been suggested for both AD and PD diagnosis in the early and prodromal stages [3, 4]. In terms of imaging, atrophy on structural magnetic resonance imaging (sMRI) or hypometabolism/hypoperfusion on PET/SPECT as a measure of neuronal injury/neurodegeneration in AD, and abnormal presynaptic dopaminergic SPECT/PET or cardiac sympathetic denervation on MIBG scintigraphy in PD, are considered to support the diagnosis (in the context of the presence of other typical diagnostic features of PD/AD). The current review focuses solely on sMRI.

Gray matter (GM) atrophy is a cardinal sign of neurodegeneration. A strong link exists between the neuropathology of neurodegeneration and GM atrophy seen on sMRI. In PD, a typical cortical GM atrophy pattern has not yet been conclusively established, although recent studies have used multivariate analytical approaches to identify specific atrophy patterns in early PD that may predict the malignant course of the disease, including the cognitive decline, e.g., [5•, 6•, 7••].In AD, prominent mesiotemporal atrophy (MTA) is typically present already in the early disease stages [8, 9] and it has been proposed as a useful biomarker in the predementia states of AD [10, 11] which can be used as a supportive diagnostic sign even on single subject level. The relative preservation of medial temporal structures on sMRI has been proposed as supportive biomarker for dementia with Lewy bodies (DLB), [12]. However, several AD variants have been described that may not show decreased hippocampal volumes (e.g., [13–15]. Moreover, recent studies demonstrate that DLB cannot be fully distinguished from AD based on AD-specific brain atrophy patterns; however, they provide information about additional AD-related brain pathology in DLB cases [16].

On the whole, it can be noted that regions typically affected by GM atrophy correlate, to a certain extent, with the cognitive profile and severity of cognitive impairment. Typical GM atrophy distribution as revealed by sMRI can increase diagnostic accuracy and thus serve as an early diagnostic biomarker especially for AD [3], and as a biomarker of disease progression and MCI and dementia prediction in both PD and AD (e.g., [5•, 9, 17•, 18•, 19, 20]). However, the situation may vary in individual subjects and may be modified by cognitive reserve (e.g., [21]). Cognitive reserve may also explain why clear cortical atrophy in regions known to be related to cognitive impairment in PD may be present in PD subjects with normal cognition [22] and why this finding may reflect a higher risk for cognitive decline over time [23].

This paper focuses particularly on the current approaches to studying GM atrophy patterns and patterns of atrophy rates/percent changes of GM in PD and AD with respect to their prediction of PD-MCI and PD/AD dementia conversion that may be relevant for treatment strategies.

## Methods Used to Evaluate Gray Matter Atrophy

In general, GM abnormalities are usually assessed using either volume-based morphometry (VBM) or surface-based analyses. Cortical thickness (CT) is designated as the distance in millimeters between white and pial surfaces. Smoothed values of CT assigned to vertices can be compared across subjects and groups. As compared to VBM, CT is more sensitive to cortex changes, possibly because it is less dependent on cortical folding and the overall brain size [22, 24].

Source-based morphometry (SBM) is a multivariate method that makes it possible to identify spatially independent sources of local GM variability that share the same covariance across subjects [25, 26]. The method can identify the patterns (components) of GM alternations and reduces the problem of multiple testing. Deformation-based morphometry (DBM) is based on the application of non-linear registration for spatial normalization [27]. The resulting deformation fields provide information about the structural differences between the analyzed brain and the template brain. DBM may be more sensitive to subcortical atrophy than other methods [28].

## Gray Matter Changes in Parkinson’s Disease and Relation to Cognition

Cognitive impairment (CI) in PD is particularly predicted by biomarker changes related to nigrostriatal or cortical dopaminergic deficits and alterations in acetylcholine and norepinephrine systems, global atrophy possibly due to widespread effects of brain degenerative processes, comorbid Alzheimer’s disease plaque pathology, and genetic factors [29]. Demographic and clinical risks for cognitive decline have also been established, including increasing age, PD duration, male sex, specific motor features (postural instability and gait difficulty phenotype), and non-motor symptoms such as REM sleep behavioral disorders, autonomic dysfunction, anosmia, apathy, depression, and hallucinations [30].

Earlier studies that focused on sMRI correlates of CI in PD used particularly VBM and revealed rather inconsistent findings, with dominating cortical atrophy in the frontal, parietal, and (mesio)temporal or occipital lobes, e.g., [26, 31–35]. The inconsistencies were caused by heterogeneous disease progression; small sample sizes; less precisely characterized PD-CI, PD-MCI, and PDD subgroups; and methodological issues. Cortical thickness analysis may better discriminate cortical morphometrical patterns and may be more sensitive to age or disease-related regional gray matter changes than VBM [22, 36]. The situation is further complicated by the fact that in PD-MCI, all cognitive domains may be impaired, with attention/executive functions deficits being the most common impairment; however, multiple-domain MCI is also common, and dominating memory deficits resembling that of AD may also occur [37].

Indeed, it is likely that the specific pattern of disease spread in PD may influence the pattern of GM atrophy and clinical features of PD-MCI and PDD. A “dual-syndrome” hypothesis was formulated [38]. According to this hypothesis, a distinction can be made between fronto-striatal (dopaminergically modulated) executive dysfunction, which may not herald the progression of serious cognitive decline, and a dementia syndrome characterized by cholinergically mediated memory and visuospatial deficits associated with posterior cortical and temporal lobe dysfunction [38, 39]. However, only well-designed longitudinal studies in early PD with the use of relevant biomarkers can provide further evidence to support this hypothesis.

More recent sMRI studies (published particularly between 2014 and 2019) aimed at identifying specific PD subtypes mostly in newly diagnosed drug-naïve well-characterized PD patients using the PPMI database (http://www.ppmi-info.org/data) and ICICLE database (PD community and outpatient clinics in northeast England), and prospectively studied their progression over time in terms of GM atrophy patterns and atrophy rates, clinical profiles, and prediction of MCI conversion, e.g., [5•, 6•, 17•, 23, 29]. The studies focused on assessing volume/cortical thickness of dopaminergic and cholinergic subcortical nuclei and their cortical projection regions [17•, 40, 41], as well as the subcortical-cortical atrophy patterns [5•], hippocampal subfields [41], and expressions of the spatial pattern of abnormality for recognizing early Alzheimer’s disease (SPARE-AD) [18•]. We summarize the results of recent works in the text below and in the Table 1 in Suppl. material.

In the studies comparing various PD subtypes, authors classified PD patients either based on their clinical syndromes (motor and non-motor/cognitive features) (e.g., [6•, 34]) or based on observed atrophy patterns derived from an exploratory cluster analysis (e.g., [42, 43]). Based on motor scores and three non-motor features (cognitive impairment, REM sleep behavioral disorder, and dysautonomia), Fereshtehnejad et al. [6•] classified the PD patients as mild-motor predominant, intermediate, and diffuse-malignant. The “diffuse malignant” PD subtype had greater cognitive decline after an average of 2.7 years and more pronounced atrophy on sMRI in PD brain networks than other subtypes, together with more prominent AD pathology in CSF, greater dopaminergic deficit as assessed by DAT imaging, and faster progression of motor and cognitive deficits. Of note, the authors made use of the single “PD-specific atrophy network” they described earlier [28], using it as a region of interest in their data analyses. This specific sMRI component is derived from DBM and independent component analysis. It was detected in a large number of early drug-naïve PD patients (*n* = 232 from PPMI dataset) as compared to HC (*n* = 117) and consists of atrophy of subcortical structures in particular, containing the basal ganglia, the pedunculopontine nucleus, and the basal forebrain, including the nucleus basalis of Meynert, hypothalamus, amygdala, hippocampus, parahippocampal gyrus, and two thalamic regions, the ventrolateral nucleus and pulvinar. Cortical regions in this network are the insula, occipital cortex, superior temporal gyrus, rostral anterior cingulate cortex, premotor and supplementary areas, and parts of the lateral prefrontal cortex [28]. Later work by the same group [5•] tested the “disease propagation hypothesis” in de novo PD patients (*n* = 105) and healthy controls (*n* = 57). The authors predicted that cortical brain atrophy at a follow-up visit (after 1 year) will depend on the connectivity of cortical areas to affected areas at baseline. Cortical regions with the greatest cortical thinning at the follow-up visit were in the limbic, frontoparietal, and ventral attention networks and anterior parts of the default mode network. Using diffusion tensor imaging and resting-state functional MRI, the authors found that cortical regions with greater connectivity to the subcortical PD-related network identified at the time of diagnosis demonstrated greater cortical atrophy over the 1-year period. This is a very interesting assumption that needs to be tested in the future with a longer follow-up period and with a deeper focus on cognitive sequelae (in the current study, only uncorrected results were available for association with MoCA scores).

Using the cortical thickness approach, Uribe et al. [42] described three patterns of cortical thinning in non-demented PD patients (with a mean disease duration of 6–8 years) as compared to controls. Pattern 1, with GM atrophy mainly in the parietal and temporal regions, was associated with worse cognitive performance. Pattern 2 had frontal and occipital GM atrophy, but the medial parietal and temporal regions were preserved. This was particularly associated with a younger age at disease onset. Pattern 3 had no detectable cortical thinning. In a later study of the same group [43], the authors described only two patterns of GM atrophy in de novo PD patients from the PPMI dataset: pattern 1 with GM atrophy mainly in the anterior brain regions (orbitofrontal and anterior temporal regions and anterior cingulate), and pattern 2 with GM atrophy mainly in the posterior brain regions (occipital and parietal regions, cuneus, postcentral gyrus) that showed cognitive impairment in neuropsychological testing (memory and other domains). Although the subjects were de novo PD patients, the authors did not find a subgroup without GM atrophy, as they had in their previous work. They explained this discrepancy by the high sensitivity of the methodological approach in the later study (improved cluster analytical approach). However, both studies are cross-sectional and may reflect the concrete rather small PD groups studied. Later, as a follow-up to the first study [42], the authors evaluated the progression of the three abovementioned atrophy subtypes over 4 years [44]. In pattern 1 (baseline temporo-parietal atrophy), two patients converted to dementia; the majority of subjects were lost to follow-up care, probably due to the more malignant course of the disease. The group was not included in the longitudinal evaluation. In the other two groups, there were several MCI patients; some were converters and some were MCI already at baseline. Unexpectedly, patients in pattern 3, non-atrophic at baseline, showed more progressive cortical thinning (in bilateral temporal and parietal regions) than patients in pattern 2, which had frontal and occipital atrophy at baseline. The patients in pattern 2 were younger with earlier disease onset and higher education; these factors might be protective against cortical thickness changes over time.

Probably most informative are longitudinal studies evaluating the progression of cognitive decline together with GM changes and comparing PD subjects with conversion to cognitive deficits and those with cognitively stable PD, e.g., [29, 45]. Probably the most consistent finding of sMRI studies is frontal atrophy in PD. Studies showing atrophy mainly in the frontal regions [5•, 46•, 47] usually found correlations between frontal lobe atrophy and worsening of frontal (executive) functions. In contrast to the “dual hit” hypothesis, this was particularly true for studies that focused on dementia prediction. In the longitudinal study by Chung et al. [46•], drug-naïve PD-MCI patients at baseline were prospectively followed for 4 years and were then categorized into dementia converters and non-converters. PD dementia converters had widespread cortical thinning extending from the posterior cortical regions to frontal areas; in PD dementia non-converters, the cortical thinning was observed in the occipital regions and precuneus, relative to controls. Neuropsychological scores of frontal functions correlated with cortical thickness in frontotemporal and posterior parietal regions. These findings suggest that cortical thinning in the frontal areas alone or in combination with more widespread brain atrophy drives the conversion to dementia in PD-MCI patients. Another study showed that PD-MCI patients who converted to dementia over time had at baseline greater atrophy in the frontal lobe, insula, and left middle temporal cortex than non-converters and showed overall more extensive cortical thinning as compared to HC and relative to non-converters [19].

Others classified early PD patients, depending on whether they developed MCI over time, as MCI converters and MCI non-converters [47, 48]. PD-MCI converters had significantly greater atrophy in the left prefrontal areas, left insular cortex, and bilateral caudate nucleus as compared with PD-MCI non-converters, and larger atrophy in the bilateral parahippocampal gyri and the left cerebellum as compared with healthy controls. A decline of executive functions in PD-MCI converters positively correlated with atrophy of the frontal regions [47]. In another study, PD-MCI converters had decreased GM as well as WM volume in the frontal and parietal regions and this GM/WM reduction correlated with cognitive decline in frontal functions across time. However, only WM atrophy of the frontal regions predicted conversion to MCI over time [48]. Comparing the amyloid-positive PD group (as measured by Aβ level in CSF) with amyloid-negative PD subjects cross-sectionally, PD Aβ+ patients had GM atrophy predominantly in the frontal cortex; PD Aβ− patients had mainly subcortical atrophy (SN and brainstem) [49]. Aβ+ PD patients were older, with higher frequency of APOE4 allele, and had lower cognitive performance than PD Aβ− patients. Yau et al. [5•] also observed an association between the left frontal and occipital cluster and CSF Aβ. However, cognitive correlates of these changes were not found [5•].

The presence of AD atrophy patterns described previously by Weintraub et al. [32], covering most of the temporal regions (especially the hippocampus, entorhinal cortex, inferior temporal lobe) and the precuneus/posterior cingulate, as well as baseline CSF Aβ, was shown to independently predict subsequent cognitive decline in PD [18•, 32]. Hippocampal atrophy has been associated with mainly memory impairment in PD. Uribe et al. [50•] segmented the hippocampus into 12 subregions and showed in a longitudinal study that changes in several hippocampal subregions are predictive for memory loss in PD patients. Foo et al. [41] found longitudinal changes in hippocampal subfields (cornu amonis 2,3) and in episodic memory in PD-MCI converters as compared to PD stable. Of note, this study did not include healthy controls, and thus, it cannot distinguish whether the changes were solely due to PD neurodegeneration or due to additional effects of normal aging. In a prospective study by Kandiah et al. [51], hippocampal volume was a major predictor for developing MCI and dementia in PD. However, the cognitive scores were not tested for correlation with hippocampal atrophy. The rationale for studying atrophy in the hippocampal subfields is that the cytoarchitecture of hippocampus is not homogeneous; it consists of several regions with distinct histological characteristics and specific connections [41]. Thus, studies of hippocampal subfields rather than those of the whole hippocampal volume may better detect subtle changes in the hippocampal structure and more precisely discriminate cognitive correlates of these changes and identify subjects at higher risk of cognitive decline.

Some authors also used advanced techniques to study the subcortical regions. The specific PD-related network described above [28] included particularly basal ganglia subcortical structures. Hanganu et al. [52] found longitudinal decreases in the volumes of the amygdala and nucleus accumbens in PD-MCI, together with faster rates of cortical thinning in the temporal, occipital, and SMA regions. MoCA scores correlated with atrophy in the temporal and medial occipital lobe in all PD patients. PD-MCI patients showed atrophy of the nucleus accumbens (together with widespread cortical thinning) compared to both HC and PD-NC in the study by Mak et al. [23]. Atrophy of the hippocampus, amygdala, thalamus, caudate nucleus, putamen, and nucleus accumbens was also found in the study by Vasconcellos et al. [53]; worse cognitive outcome in PD was correlated with reduced volume of these structures.

Recent developments in stereotactic mapping of the basal forebrain nuclei made this region accessible to detailed MRI-based morphometric assessments. Ray et al. [17•] found that smaller volumes of the nucleus basalis of Meynert in early PD were associated with greater change in global cognitive scores, but not motor scores, after 2 years. Using an arbitrary threshold of 1 SD below the control range, the authors demonstrated that PD with smaller volumes had 3-fold elevated risk of developing MCI or PDD. Cholinergic deficits seem to be crucial for the development of dementia and the spatial congruence of cholinergic deficits, and brain hypometabolism argues for cortical deafferentation due to the degeneration of projection fibers from the basal forebrain [54].

## Gray Matter Changes in Alzheimer’s Disease and Relation to Cognition

Atrophy, as a measure of neurodegeneration and brain injury, is related to tau accumulation in certain brain regions and is correlated to in vivo biomarkers of tau accumulation and brain hypometabolism such as cerebrospinal fluid (CSF) p-tau/total tau levels, PET imaging with tau ligands, or fluorodeoxyglucose (FDG) PET. The process of neurodegeneration starts long before clinical symptoms manifest. In this period, subtle cognitive and neuropsychiatric disturbances can be present [55] concomitant with the presence of atrophy on sMRI. Certain sMRI changes in specific brain regions thus can be detected up to 10 years before clinical diagnosis of AD [56], particularly atrophy of medial temporal regions and the hippocampus in typical AD. Recent sMRI studies results are summarized also in the Table 2, in Suppl. material.

Hippocampal atrophy on sMRI is probably the best documented specific pathological feature in AD and has a potential to differentiate prodromal AD subjects from healthy subjects as well as from other pathological conditions. NIA-AA proposed new research criteria for AD [57] in 2018 with sMRI proof of GM atrophy as one of the biomarkers of neurodegeneration. With the new approach to assessing hippocampal subfield atrophy, it is possible to better identify very early changes related to pathological aging and AD. It seems that the cornu amonis (region 1 especially) and subiculum are the most vulnerable to degeneration in AD as well as in aging [58].

“Typical” AD, with prominent memory impairment, represents about 60% of all AD cases. The characteristic feature is medial temporal atrophy (MTA) together with atrophy of other temporal regions and posterior cingulate/precuneus atrophy (often referred to as the AD atrophy pattern) [32, 59]. The “atypical” AD cases split into AD with prominent visual impairment (posterior cortical atrophy—PCA), AD with prominent language problems (logopenic primary progressive aphasia—lvPPA), and the behavioral/dysexecutive variant of AD. While the pattern of amyloid distribution remains similar in all AD subtypes, the distribution of tau and the pattern of GM atrophy vary. The main regions affected by atrophy/neurodegeneration in PCA include the occipital, parietal, and occipito-temporal regions [13]; in lvPPA, the affected regions are the posterior portions of the superior and middle temporal lobe, the inferior parietal lobe, and the temporo-parietal junction [14]; in the behavioral/dysexecutive variant of AD, the atrophy pattern is similar to that of typical AD [60]. Neurodegeneration patterns may be asymmetrical in atypical AD, especially in lvPPA [15].

Studies comparing the various clinical subtypes of AD have classified the patients either based on their clinical characteristics or based on distinct atrophy patterns. Based on atrophy patterns, Park et al. [61•] classified three atrophy subtypes: parietal predominant (P), medial temporal predominant (MT), and diffuse (D). The P subtype had an earlier disease onset and worse cognitive performance in all areas except for verbal memory tests. The MT and D subtypes had similar cognitive profiles except for the language and visuospatial domains, in which the MT subtype had worse performance. Based on neuropsychological performance, Scheltens et al. [62•] categorized probable AD dementia patients into an AD subtype with prominent memory impairment and a non-memory AD subtype. The non-memory cohort had more severe posterior atrophy and relatively spared hippocampi. Firth et al. [63•] longitudinally followed patients with PCA who showed early occipital and parietal atrophy, with subsequent higher rates of temporal atrophy (hippocampal, entorhinal, and frontal atrophy underwent a lower rate of progression). In contrast, patients with typical AD showed early hippocampal atrophy, with subsequent higher rates of temporal atrophy. Visuospatial cognition declined earlier in PCA; working memory declined earlier in typical AD.

The atrophy pattern also varies depending on the age and the disease stage. Lee et al. [64•] studied atrophy at different stages across the AD continuum (cognitively normal, subjective cognitive impairment, early amnestic MCI, late amnestic MCI, very mild AD, mild AD, and moderate/severe AD) to investigate the differences between physiological and pathological aging. Their results suggest that cortical thinning in the precuneus and inferior temporal regions in AD continuum subjects differentiates between pathological and physiological aging. In preclinical AD, cortical thickness was not associated with cognition, but in the clinical stages of AD (prodromal and probable AD), the tau PET imaging and cortical thickness showed strong cognitive correlates (in a variety of neuropsychological tests), mostly with thinning in the lateral and medial parietal cortex and lateral temporal cortex [65]. Structural MRI predicted AD diagnosis (prodromal or probable AD) with slightly lower accuracy against tau PET but with similar regions affected by neurodegeneration (amygdala, entorhinal cortex, parahippocampal gyrus, hippocampus, fusiform gyrus, and inferior parietal lobule) that was also strongly associated with cognitive outcome [66].

Finally, sMRI results were closely related to the postmortem distribution of neurofibrillary tangles according to a clinico-pathological study of Whitwell et al. [67]. Based on a pathological classification of atypical AD variants, hippocampal sparing (HpSp) AD shows minimal involvement of the hippocampus while limbic predominant (LP) AD shows neurofibrillary tangles only in the medial temporal lobe. The proportion of subjects who presented with an amnestic syndrome was lowest in HpSp AD (42%) and highest in LP AD (94%). As for antemortem sMRI findings, typical AD showed gray matter loss throughout medial temporal lobe and lateral temporoparietal cortex, with additional involvement of the precuneus, insula, medial and lateral frontal lobe, and caudate nucleus, compared to controls. HpSp AD revealed a relative sparing of the medial temporal lobe, with loss predominantly in the temporoparietal cortex, insula and precuneus, frontal lobe, and caudate nucleus. GM loss in LP AD was in the medial temporal lobe, particularly involving the hippocampus, amygdala, and entorhinal cortex. Based on pathological evaluations, a novel sMRI marker was described that provided the best discrimination across all three AD subtypes, i.e., the ratio of hippocampal-to-cortical volume. A later study of Risacher et al. [68••] showed that an increased hippocampus-to-cortex ratio in AD patients predicted faster clinical decline in AD patients who were clinically indistinguishable at baseline except for a greater dysexecutive syndrome.

## Conclusions and Future Directions

In PD, recent sMRI studies show that the dual hypothesis [38] may not be generalizable and it does not work for all PD subjects. Studies combining dopaminergic SPECT with sMRI demonstrated that dopaminergic denervation is associated with frontal as well as posterior cortical atrophy [69]; this is in accordance with the known structural and functional connectivity of the basal ganglia [70, 71], and frontal lobe atrophy alone predicts dementia in the PD population [46•]. Novel methodological approaches made it possible to study cholinergic nuclei losses in the basal forebrain and have shown that they are present early in the disease course, probably leading to atrophy of cortical projection areas both in the prefrontal and posterior cortical regions [72] and to the subsequent progression of cognitive deficits [17•]. Future studies should verify these results by utilizing combined cholinergic PET/sMRI biomarkers in the same PD population.

The AD-related atrophy pattern is expressed already in early PD subjects and leads to cognitive decline over time. It may not be fully interpreted by the spread of β-amyloid pathology alone [18•], but future studies that combine amyloid/tau PET and sMRI are warranted to shed further light on this assumption. Future studies should also explore how cholinergic neuron losses in the nucleus basalis of Meynert and other cholinergic structures of the basal forebrain impact the SPARE-AD pattern of atrophy in PD and the progression of memory and visuospatial deficits in PD [73]. Further studies exploring the effect of atrophy on distinct hippocampal subfields are also awaited.

Finally, a single PD-related pattern (component) that contains both subcortical structures and cortical brain regions was identified using a novel analytical approach: DBM in combination with independent component analysis [5•, 28]. Future studies should determine whether this complex subcortico-cortical PD-related pattern in early PD may underlie progression of cognitive deficits and identify the exact cognitive profile associated with this pattern and its changes over time. While preliminary but exciting results have been published using the partial least square (PLS) analysis and combining clinical and various biomarkers with the use of PD-related pattern [7••], studies are needed with a longer follow-up to show whether this or other relevant patterns of brain atrophy in early PD may predict fast PD-dementia conversion which would be of therapeutic relevance.

As for AD, the Aβ deposition in the cortex of AD patients is rather diffuse and seems to be uniform, not reflecting the clinical heterogeneity of AD. As a biomarker of neuronal loss, sMRI follows the spatial distribution of tau and correlates better with clinical symptoms of particular AD variants. The hippocampus-to-cortex ratio seems to be the best sMRI biomarker to discriminate between various AD subtypes, closely following the spatial distribution of tau pathology in distinct Murray-Dickson AD subtypes. Combinatorial sMRI, CSF/PET, and neuropsychological longitudinal prospective studies in preclinical and prodromal populations (subjective cognitive impairment, mild cognitive impairment, amnestic and non-amnestic) as compared to healthy aging populations are needed to evaluate the ability of the hippocampus-to-cortex ratio and other specific cortical patterns in the early identification of distinct typical and atypical forms of AD and predict the precise phenoconversion and disease course. The impact of age, sex, and genetic factors alongside cognitive reserve and its manipulation [21, 74] should also be further explored.

## Electronic Supplementary Material

ESM 1 (DOCX 29 kb)
