# Supplementary material for: Gray Matter Changes in Parkinson’s and Alzheimer’s Disease and Relation to Cognition
Source: Curr Neurol Neurosci Rep. 2019 Nov 13;19(11):85. doi: 10.1007/s11910-019-1006-z (PMC6854046; doi:10.1007/s11910-019-1006-z)
Supplement: Supplementary file 1 — (DOCX 29 kb) [file 11910_2019_1006_MOESM1_ESM.docx]

**Table S1: Selected recent structural MRI studies regarding PD**

| **Study** | **Subjects** | **Methods** | **Outcomes** | **Main results** |
| --- | --- | --- | --- | --- |
| Caspell-Garcia et al., 2017 | 160 PD | Brain parcellation using Freesurfer longitudinal pipeline | The finding of baseline and longitudinal predictors of cognitive impairment over the 3-year time period | PD: smaller baseline fusiform, lateral occipital and lateral orbitofrontal volumes predicted cognitive impairment |
| Duncan et al., 2016 | 125 *de novo* PD, 50 HC | VBM using SPM | GM volume: PD vs HC, correlation with cognitive scores | PD vs HC: no difference in GM volume PD: bilateral reductions in frontal and parietal GM volume associated with worse executive functions |
| Fereshtehnejad et al., 2017 | 223 "mild motor-predominant" PD, 52 "diffuse malignant" PD, 146 "intermediate" PD | Independent component analysis (ICA) on DBM maps | Atrophy examination in Parkinson's disease-specific network: HC vs PD subtypes | "Diffuse malignant" PD subtype had more atrophy and "mild motor-predominant" PD subtype had least atrophy in the Parkinson's disease-specific brain network |
| Foo et al., 2016a | 54 PD-NC, 11 PD-MCI | Subcortical structures and hippocampal subfields segmentation using longitudinal Freesurfer pipeline | Subcortical structures volume: PD-MCI vs PD-NC, PD (converters) vs PD (stable), change over time (1 year), correlations with cognitive scores | PD-MCI vs PD-NC: greater baseline thalamus atrophy and progressive atrophy in the thalamus, caudate, presubiculum, cornu ammonis 1 and 2-3 PD (converters) vs PD (stable): greater accumbens atrophy at baseline and progressive atrophy in the thalamus, caudate and accumbens |
| Foo et al., 2016b | 54 PD-NC, 11 PD-MCI | Hippocampal subfields segmentation using longitudinal Freesurfer pipeline | Hippocampal subfields volume: PD-MCI vs PD-NC, PD (converters) vs PD (stable), change over time (1 year), correlations with cognitive scores, logistic regression to predict conversion | PD-MCI vs PD-NC: lower volumes in the left fimbria, right CA1 (correlated with attention), and right HATA Decline in volumes of CA2-3 in PD (converters) after 18 months when compared to PD (stable) Baseline volumes of GC-DG, right CA4, left parasubiculum and left HATA were predictive of the conversion from PD-NC to MD-MCI |
| Garsia-Diaz et al., 2016 | 92 PD, 36 HC | Cortical thickness evaluation using Freesurfer | Cortical thickness: PD vs HC, correlations with cognitive scores; PD (normal pentagon item scores) vs PD (abnormal pentagon item scores) | PD vs HC: lower cortical thickness in bilateral temporo-parietal-occipital regions, increased lateral ventricular volume and reduce cortical thickness in PD PD: correlation between MMSE and global atrophy measures PD (normal pentagon item scores) vs PD (abnormal pentagon item scores): cortical thickness reductions in bilateral temporo-parietal-occipital regions in PD (abnormal pentagon item scores) |
| Garsia-Diaz et al., 2018 | 28 PD-NC, 16 PD-MCI, 20 HC | Cortical thickness evaluation using longitudinal Freesurfer pipeline | Symmetrized percent change of cortical thickness (after 4 years): PD-NC vs PD-MCI, HC vs PD-NC, HC vs PD-MCI; correlations with neuropsychological data | PD-MCI vs PD-NC: greater progressive cortical thinning in left lateral occipital and inferior parietal regions, and in right medial temporal regions in PD-MCI PD-MCI: correlation between the change in visuospatial and visuoperceptual functions and symmetrized percent change of cortical thinning in occipital, parietal and temporal regions PD-NC: correlation between Facial Recognition test and thinning in parieto-occipital regions |
| Gasca-Salas et al., 2019 | 23 PD-MCI, 18 HC | Cortical thickness evaluation using Freesurfer | Identification of patterns of regional atrophy in PD-MCI who developed dementia during 31 months of follow-up (n=8) | PD-MCI (converters) vs PD-MCI (non-converters): cortical thinning bilaterally in frontal, insula and the left middle temporal areas |
| Hanganu et al., 2014 | 15 PD-NC, 17 PD-MCI, 18 HC | Cortical thickness evaluation using longitudinal Freesurfer pipeline | Cortical thickness: rate of change (19.8 months), correlation with cognitive scores | PD-MCI vs HC: faster rate of cortical thinning in the temporal lobe, supplementary motor area and medial occipital lobe, loss of volume of amygdala and nucleus accumbens PD-NC vs HC: faster rate of cortical thinning in the lateral occipital lobe MoCA scores correlated with cortical thinning over time in PD |
| Chung et al., 2019 | 34 *de novo* PDD (converters), 34 *de novo* PDD (non-converters) | Cortex surface reconstruction using various algorithms and cortical thickness evaluation | Cortical thickness: PDD (converters) vs PDD (non-converters), HC vs PDD (converters), HC vs PDD (non-converters); prediction of PDD conversion within 4 years using Linear Discriminant Analysis (LDA) | PDD (non-converters) vs HC: reduced cortical thickness in the bilateral occipital and precuneus regions PDD (converters) vs HC: reduced cortical thickness in the bilateral occipital, left orbitofrontal, right inferomedial temporal, bilateral occipital frontoparietal operculum, superior temporal, precuneus and cingulum regions PDD (converters) vs PDD (non-converters): reduced cortical thickness in the bilateral medial frontal, left orbitofrontal, right inferomedial temporal, bilateral occipital, frontoparietal operculum, superior temporal and cingulum regions LDA: prediction model with cortical thickness in the medial part of the right superior frontal gyrus and left olfactory cortex optimally distinguished PDD (converters) from PDD (non-converters) |
| Kandiah et al., 2014 | 71 PD and 26 PD-MCI (44 PD-NC, 34 PD-MCI, 8 PDD after 2 years) | Hippocampal segmentation using FSL-FIRST | Identification of MCI and dementia conversion predictors using logistic regression analyses | Hippocampal volume was a significant predictor for MCI and dementia |
| Kunst et al., 2018 | 23 PD-NC, 24 PD-MCI, 58 HC | VBM using SPM, SoBM, cortical thickness evaluation using Freesurfer | Cortical thickness: PD-NC vs PD-MCI, HC vs PD-NC, HC vs. PD-MCI, correlations with cognitive scores | PD-MCI vs PD-NC: greater GM atrophy in the orbitofrontal regions - correlation with memory scores, and in left superior parietal lobule PD-MCI vs HC: widespread limbic and fronto-parieto-occipital neocortical atrophy PD-NC vs HC: subtle GM atrophy in the anterior cingulate, precuneus and temporal neocortex |
| Lee et al., 2014 | 15 PD-MCI (converters), 36 PD-MCI (non-converters), 25 HC | VBM using SPM and volumetric analysis of substantia innominata (SI) | GM density and SI volume: PD-MCI (converters) vs PD-MCI (non-converters), HC vs PD-MCI (converters), HC vs PD-MCI (non-converters) | PD-MCI (non-converters) vs HC: lower GM density in the right middle frontal cortex and bilateral parietal areas, smaller mean normalised SI volume PD-MCI (converters) vs HC: lower GM density in the insular cortex, extending into inferior frontal area, bilateral parahippocampal gyrus, left cerebellum and left caudate, smaller mean normalised SI volume PD-MCI (converters) vs PD-MCI (non-converters): lower GM density in the left frontal areas, left insular cortex and bilateral caudate nucleus, smaller mean normalised SI volume |
| Lorio et al., 2019 | 205 PD, 105 HC | VBM using SPM | GM volume: PD vs HC, covariance analysis with DAT-SPECT | PD vs HC: no significant differences, association between DAT-SPECT signal in the putamen and GM volume in the left prefrontal, premotor cortex and the insula (positive correlation in PD, negative correlation in HC) |
| Mak et al., 2015 | 66 PD-MC, 39 PD-MCI, 37 HC | Cortical thickness evaluation using longitudinal Freesurfer pipeline | Cortical thickness: PD-NC vs PD-MCI, HC vs PD-NC, HC vs. PD-MCI, longitudinal analysis (18 months),correlations with cognitive scores | Baseline: PD-MCI demonstrated widespread cortical thinning relative to HC and atrophy of nucleus accumbens compared to both HC and PD-NC  cortical thickness in frontal and temporo-praietal cortices correlated with global cognition in PD   PD-NC (converters) showed bilateral temporal cortex thinning when compared to PD-NC (stable) Follow-up: PD-MCI demontrated more severe cortical thinning in frontal and temporo-parietal cortices, and hippocampal atrophy, in comparison to HC and PD-NC  PD-NC showed more severe frontal cortical thinning in comparison to HC |
| Mak et al., 2017 | 64 PD-NC, 36 PD-MCI, 38 HC | Evaluation of whole-brain atrophy using FSL-SIENA and ventricular enlargement using VIENA | Percentage brain volume change and ventricular enlargement: PD-NC vs PD-MCI, HC vs PD-NC, HC vs PD-MCI | PD-MCI: increased global atrophy and ventricular enlargement over 18 months in comparison to both PD-NC and HC |
| McMillan et al., 2016 | 61 *de novo* amyloid positive PD (PD+), 308 *de novo* amyloid negative PD (PD-), 174 HC | VBM using FSL | GM volume: PD vs HC, PD+ vs HC, PD- vs HC | PD+ vs HC: bilateral atrophy in the medial and lateral frontal, temporal and parietal cortex PD- vs HC: atrpohy in medial and lateral cortex, thalamus and putamen and moderate right lateral frontal cortex disease PD+ vs PD-: atrophy in medial and lateral frontal regions, thalamus and middle temporal cortex PD- vs PD+: atrophy in bilateral substantia nigra, brainstem and superior medial parietal cortex |
| Nedelska et al., 2015 | 20 autopsy confirmed DLB, 22 mixed DLB/AD, 30 AD, 15 HC | Atrophy estimation using TBM | Atrophy rates calculation (longitudinal follow-up until death) and comparison across groups | DLB without AD-type pathology: lower global and regional rates of atrophy, similar to HC Mixed DLB/AD: greater rates in the whole brain, temporo-parietal cortices, hippocampus, amygdala and ventricle expansion, similar to AD Correlation of atrophy rates with Braak neurofibrilary tangle stage, cognitive decline and progression of motor symptoms |
| Noh et al., 2014 | 28 *de novo* PD-NC, 24 *de novo* PD-MCI | VBM using SPM | GM volume: PD-NC vs HC, PD-MCI vs HC, PD-MCI vs PD-NC | PD-NC vs HC: GM volume loss in the right hippocampus, right cuneus and right precuneus PD-MCI vs HC: GM volume loss in bilateral temporal and frontal areas PD-MCI vs PD-NC: GM volume loss in the right temporal pole, left precuneus, medial frontal and posterior cingulate gyrus |
| Pereira et al., 2014 | 90 PD-NC, 33 PD-MCI, 56 HC | Cortical thickness evaluation using Freesurfer | Cortical thickness: PD-NC vs HC, PD-MCI vs HC, PD-MCI vs PD-NC, correlations with cognitive scores | PD-NC vs HC: cortical thinning in right inferior temporal gyrus, correlation with verbal learning scores PD-MCI vs HC: cortical thinning in right inferior temporal gyrus, left superior parietal cortex, precuneus, lateral occipital, temporal, anterior cingulate and superior frontal gyri PD-MCI vs PD-NC: cortical thinning in left precuneus Memory, executive and visuospatial functions associated with widespread cortical atrophy |
| Ray et al., 2018 | 168 *de novo* PD, 76 HC | VBM within ROIs using SPM | Identification of PD with smaller cholinergic basal forebrain volumes, binary classification, regression, survival analysis, linear mixed modelling | PD: smaller volumes of nucleus basalis of Meynert were associated with greater change in global cognitive scores after 2 years patients, patients with smaller volumes had ~3.5-fold greater risk of being categorized as PD-MCI and they showed more severe and rapid decline in memory and semantic fluency over a period of up to 5 years |
| Rektorova et al., 2014 | 75 PD-NC, 29 PD-MCI, 22 PDD, 25 HC | Source based morphometry | Two patterns of grey volume deviations identification, correlations with cognitive scores | Pattern 1: reductions in hippocampus and temporal lobes Pattern 2: decreases in fronto-parietal regions and increases in the midbrain/cerebellum Both patterns differentiated PDD from all other groups and correlated with visuospatial deficits and letter verbal fluency Pattern 2 differentiated PD-NC from HC |
| Sampedro et al., 2019 | 87 *de novo* PD, 38 HC | Cortical thickness evaluation using longitudinal Freesurfer pipeline | Symmetrized percent change in cortical thickness, correlations with clinical parameters | Reduced DAT uptake in PD patients was associated with cross-sectional and longitudinal cortical thinning in frontal and posterior cortical brain regions Imaging parameters correlated with cogitive indicators in multiple domains that extend beyond frontal-executive tasks Dopaminergic madication attenuated the longitudinal loss of cortical integrity in frontal and subset of parietal regions |
| Segura et al., 2014 | 43 PD-NC, 47 PD-MCI, 32 HC | Cortical thickness evaluation using Freesurfer | Cortical thickness: PD-NC vs PD-MCI, HC vs PD-NC, HC vs. PD-MCI, correlations with cognitive scores | PD-MCI vs HC and PD-NC: decreased total mean thickness, total GM volume, increased mean lateral ventricle volume PD-MCI vs HC: cortical thinning in bilateral parietal, temporal and occipital cortices, left superior and rostral middle areas PD-MCI vs HC: cortical thinning in bilateral superior parietal regions PD-MCI vs PD-NC: cortical thinning in right precuneus and supramarginal regions PD: cortical thinning in parietotemporal regions and less in frontal regions correlated with all neuropsychological tests |
| Uribe et al., 2016 | 88 PD, 31 HC | hierarchical cluster analysis in PD with cortical thickness in every vertex | Identification of two cortical atrophy patterns, HC vs PD cortical atrophy subtypes comparison | Pattern 1 PD vs HC: cortical thinning in bilateral precentral gyrus, inferior and superior parietal lobules, cuneus, posterior cingulate and parahippocampal gyrus; worse cognitive performance Pattern 2 PD vs HC: cortical thinning in occipital, frontal and superior parietal areas; PD with younger age at onset Pattern 3 PD vs HC: no detectable cortical thinning |
| Uribe et al., 2018a | 77 *de novo* PD, 50 HC | hierarchical cluster analysis in PD with mean cortical thickness in 360 HCP Parcellation atlas areas | Identification of two cortical atrophy patterns, HC vs PD cortical atrophy subtypes comparison | Pattern 1 PD vs HC: cortical thinning in bilateral orbitofrontal, anterior cingulate, lateral and medial anterior temporal gyri Pattern 2 PD vs HC: cortical thinning in bilateral occipital gyrus, cuneus, superior parietal gyrus and left postcentral gyrus; cognitive impairment in memory and other cognitive domains |
| Uribe et al., 2018b | 28 PD-NC, 16 PD-MCI, 21 HC | Whole hippocampus and hippocampal subfields segmentation using Freesurfer, changes over time and differences between groups assessed using repeated measures analyses | Whole-hippocampal and hippocampal subfields volume change over time (4 years), multiple regression analysis with memory scores | Significant cornu ammonis 1 reductions over time in all groups Time effect in right whole-hippocampal volume both PD-NC and PD-MCI Time effects in left hippocampal tail and right parasubiculum in PD-NC Changes in several hippocampal subregions showed predictive value for memory loss in PD |
| Uribe et al., 2019 | 45 PD, 22 HC | Cortical thickness evaluation using longitudinal Freesurfer pipeline | Symmetrized percent change of cortical thickness (after 3.8 years): investigation of cortical atrophy patterns evolution over time | Pattern 2: cortical thinning in left parahippocampal gyrus, left precuneus, right inferior parietal and temporal gyri, fusiform and lateral occipital gyri Pattern 3: cortical thinning in bilaterally in lateral and medial regions of the temporal and parietal lobes, lateral occipital and extending to frontal regions HC: cortical thinning in posterior regions Pattern 3 vs Pattern 2: more significant decrements in the right lateral occipital, lingual and pericalcarine gyri  Pattern 3 vs HC: more cortical thinning in the left pars opercularis and precentral gyri |
| Vasconcellos et al., 2018 | 48 PD, 60 HC | Subcortical structures segmentation using longitudinal Freesurfer pipeline | PD vs HC: subcortical structures volume, WM and GM volume of brain and cerebellum | PD vs HC: reduced volumes of thalamus, caudate nucleus, putamen, hippocampus, amygdala, accumbens, corpus callosum and cerebral GM |
| Wen et al., 2015 | 12 PD-MCI (converters), 30 PD-NC (non-converters) | VBM using SPM | GM and WM volume: PD-MCI (converters) vs PD-NC (non-converters), interaction between group and time (1.5 years), correlations with cognitive scores | PD-MCI (converters) vs PD-NC (non-converters): more longitudinal reduction in WM, but not GM volume, decreased WM volumes localised mainly in the frontal areas, baseline GM and WM volumes of the frontal and parietal regions associated with frontal cognitive changes across time |
| Yau et al., 2018 | 105 PD, 57 HC | Cortical thickness evaluation using CIVET | Cortical thickness: PD vs HC - longitudinal analysis (one year), correlations with functional and structural connectivity to the "disease reservoir" | Regional cortical thinning after one year greater in PD than HC in left occipital and inferior and middle temporal gyri, left frontal, right frontal and right precentral and postcentral gyrus and supramarginal gyrus. The atrophy at follow-up correlated with functional and structural connectivity of cortical areas with "disease reservoir" at the baseline. |
| Ye et al., 2017 | 207 PD (150 PD-MCI, 57 PD-NC) | Cortex surface reconstruction using various algorithms and cortical thickness evaluation | Cognitive risk score system creation and vertex-by-vertex correlation of cognitive risk score with cortical thickness | Higher cognitive risk score correlated with cortical thinning in bilateral posterior cingulate cotrex, right anterior cingulate cortex, left parahippocampal gyrus and right superior frontal cortex |
| Zeighami et al., 2015 | 232 PD, 117 HC | Independent component analysis (ICA) on DBM maps | Identification of Parkinson's disease-specific atrophy pattern (PD vs HC comparison of ICA components) | PD-specific atrophy pattern: atrophy in the midbrain, basal ganglia, basal forebrain, medial temporal lobe and discrete cortical regions; the degree of atrophy reflected clinical measures of disease severity and functional and anatomical proximity of brain regions to substantia nigra |
| Zeighami et al., 2019 | 229 *de novo* PD | Atrophy estimation using DBM | Identification of atrophy network associated with clinical phenotype using PLS | Atrophy pattern: lower brainstem, substantia nigra, basal ganglia, cortical areas consistent with Braak hypothesis |

*red: cross-sectional studies, green: longitudinal studies*

**Table S2: Selected structural MRI studies regarding AD since 2014**

| Study | Subjects | Methods | Outcomes | Main results |
| --- | --- | --- | --- | --- |
| Blanc et al., 2016 | 27 prodromal AD, 33 HC | VBM using SPM | GM volume: prodromal AD vs HC | Prodromal AD vs HC: bilateral GM loss in the hippocampal, frontal, and middle temporal gyrus, and in the posterior and mid-cingulate regions |
| Dicks et al., 2019 | 100 preclinical AD, 288 prodromal AD, 135 AD dementia | Regional AAL atlas GM volumes calculation using various softwares | Evaluation of three models taking time, age or MMSE as predictor for change in GM volume using LME | Prodromal AD vs preclinical AD: similar atrophy patterns (modeled with time and age), steeper atrophy in the right inferior temporal gyrus (modeled with MMSE) AD dementia vs prodromal AD: faster atrophy mostly in temporal lobes (modeled with time), slower atrophy in mostly frontoparietal areas (modeled with age), less steep atrophy temporal areas (modeled with MMSE) |
| Eckerstrom et al., 2018 | 24 early onset AD, 39 late onset AD, 25 younger HC, 57 elder HC | Cortical and subcortical structures segmentation using Freesurfer | Lobar and hippocampal volumes: early onset AD vs younger HC, late onset AD vs elder HC, early vs late onset AD | Hippocampal atrophy is the most prominent feature of both early and late onset AD compared with HC No significant differences between early and late onset AD |
| Fan et al., 2018 | 16 AD, 25 aMCI, 39 HC | Cortical thickness evaluation using Freesurfer | Correlations of cortical thickness with plasma levels of biomarkers and amyloid deposition (high - PiB+ group, low - PiB- group) | Mean cortical thickness was positively correlated with the plasma Aβ_40_, and negatively correlated with the plasma tau level in both PiB+ and PiB- groups |
| Fiford et al., 2018 | 153 AD, 339 MCI, 191 HC | Voxel-wise statistics on volume-change data using SPM | Group differences in age-atrophy relationship | Younger AD had faster atrophy rates in the bilateral precuneus, parietal and superior temporal lobes |
| Firth et al., 2019 | 117 posterior cortical atrophy AD, 106 typical AD, 138 HC | Construction of longitudinal trajectories of regional brain volumes | Comparison of the order in which regional brain volumes become abnormal within groups (using up to 6 annual assessments) | Posterior cortical atrophy AD: early occipital and parietal atrophy and ventricular expansion, earlier visuospatial functions decline Typical AD: early hippocampal atrophy, with subsequent higher rates of temporal atrophy and ventricular expansion, earlier working memory decline |
| Gispert et al., 2015 | 21 AD, 28 MCI due to AD, 18 preclinical AD, 62 HC | VBM using SPM and VBM8 toolbox | AD-CSF index association to brain atrophy, identification of volumetric differences associated to APOE4 | The atrophy pattern associated with the AD-CSF index was highly symmetrical and correspondended linearly with the typical AD signature The bilateral parahippocampal cortices and a parietotemporal region extending from the middle temporal to the supramarginal gyrus presented nonlinear relationship to the AD-CSF index an initial increase in volume, which later reverted APOE4 carriers showed steeper hippocampal volume reductions with AD progression |
| Hwang et al., 2016 | 77 AD, 42 HC | Cortex surface reconstruction using various algorithms and cortical thickness evaluation | Identification of 3 cortical thinning patterns, PET and CSF analyses | Medial temporal: bilateral medial temporal lobes atrophy, more glucose hypometabolism in the left hippocampus and bilateral frontal cortices and less performance in memory tests Diffuse: atrophy in nearly all association cortical areas Parietal dominant: bilateral parietal lobes and some bilateral occipital lobes, youngest, represented more glucose hypometabolism in the parietal and occipital cortices and marked amyloid-beta accumulation in most brain regions |
| Joko et al., 2016 | 58 AD, 33 aMCI, 22 HC | hippocampal atrophy index calculation using coronal slices perpendicular to the hippocampus longitudinal axis | Hippocampal atrophy index: differences between groups | Overall trend in the corrected volumes of hippocampus AD<aMCI<HC Atrophy in all slices in AD, atrophy centered on the hippocampal head in aMCI |
| Kalin et al., 2017 | 23 aMCI (non-converters), 10 MCI (converters), 23 HC | Subcortical structures and hippocampus segmentation, striatal and thalamic shape analysis; cortical thickness evaluation using CIVET | Differences between groups, ROC analysis for subcortical shape alterations | aMCI (non-converters) vs HC: bilateral striatal volume reductions aMCI (converters) vs HC: shape alterations in striatal and thalamic regions, left hemispheric morphometric changes - cortical thinning in medial temporal regions, hippocampal total and subfield atrophy at baseline with progression to similar right hemispheric alterations at time of conversion |
| Kate et al., 2018 | 299 AD (discovery data), 181 AD and 227 AD (validation datasets) | Cluster analysis using 1024 subcortical and cortical GM segmentations | Four atrophy subtypes identification and description | Subtype 1: medial-temporal predominant atrophy, worst memory and language functions, older age, lowest CSF tau levels and highest amount of vascular lesions Subtype 2: parieto-occipital atrophy, poor executive/attention and visuospatial functions and high CSF tau Subtype 3: mild atrophy, best cognitive performance, young age, highest CSF tau levels Subtype 4: diffuse cortical atrophy, intermediate features |
| Kunst et al., 2018 | 12 AD, 27 aMCI, 58 HC | VBM using SPM, SoBM, cortical thickness evaluation using Freesurfer | Differences between groups, correlations with cognitive scores | AD vs aMCI/HC: major hippocampal and temporal lobe atrophy and to some extent occipital atrophy aMCI vs HC: GM atrophy in the anterior and posterior cingulate, temporo-fronto-parietal component and in the posterior cortical regions and frontal region Correlations with cognitive deficits only in the AD group |
| Lee et al., 2019 | 874 subjective memory impairment (SMI), 954 aMCI, 847 AD, 2823 HC | Cortex surface reconstruction using various algorithms and cortical thickness evaluation | Differences between groups | AD continuum patients: relatively preserved precuneus and inferior temporal regions, decreased cortical thickness in the perisylvian region, widespread cortical thinning including precuneus and inferior temporal regions in the late-stage aMCI and AD AD continuum patients aged over 80 years: prominent cortical thinning in the medial temporal region with relative sparing of precuneus |
| Ma et al., 2016 | 15 AD, 23 MCI, 51 HC | VBM and cortical thickness evaluation | GM volume and cortical thickness: AD vs MCI | AD vs normal controls: obvious GM volume loss and cortical thinning in the right hippocampus and entorhinal cortex MCI vs normal controls: GM volume decline in bilateral hippocampus and right entorhinal cortex HC vs normal controls: slight cortical thinning |
| Mak et al., 2015 | 23 AD, 33 HC | Cortical thickness evaluation using longitudinal Freesurfer pipeline | Percent change of cortical thickness, difference in subcortical structures volume (after 1 year): AD vs HC | AD vs HC: hippocampal atrophy, lateral ventricular expansion |
| Ossenkoppele et al., 2015 | 53 early onset AD, 42 late onset AD | VBM using SPM and VBM8 toolbox | Associations between CSF biomarkers and brain atrophy in each AD variant | Relationship between lower CSF-Aβ42 and syndrome specific atrophy including precuneus, posterior cingulate and medial temporal lobe in early onset AD, and medial temporal lobe, thalamus and temporal pole in late onset AD CSF-tau was not related to GM atrophy in any group |
| Ossenkoppele et al., 2019 | 33 preclinical AD, 25 prodromal AD, 48 probable AD dementia | Cortical thickness evaluation using Freesurfer | Differences between groups, correlations with cognitive scores | Combined prodromal AD and AD dementia group: both increased [^18^F] flortaucipir uptake and reduced cortical thickness associated with worse performance on a variety of neuropsychological tests, the strongest effects were found in the lateral and medial parietal cortex and lateral temporal cortex |
| Park et al., 2017 | 225 AD, 320 HC (discovery data), 131 AD, 158 HC (validation dataset) | Cluster analysis utilizing graph theory | Three AD atrophy subtypes identification and description | Parietal predominant subtype: worst clinical presentation troughout the cognitive domains Medial temporal-predominant subtype: mild clinical presentation troughout the cognitive domains, more impaired language and executive function compared to the diffuse atrophy subtype Diffuse atrophy subtype: mild clinical presentation troughout the cognitive domains |
| Perrson et al., 2017 | 123 mild AD | Cerebral atrophy evaluation by neuroradiologist | Categorization of AD into three known subtypes, differences in cognitive scores, clinical features and progression rates | Typical AD (48%), limbic-predominant AD (24%), hippocampal-sparing AD (15%), minimal atrophy AD (13%) no differences between subtypes regarding cognitive tests results or progression rates Patients in the minimal-atrophy group were less educated, had a lower baseline CDR sum of boxes score and had higher levels of amyloid β in CSF |
| Pettigrew et al., 2017 | 232 HC at baseline, 48 developed MCI or dementia | Cortical thickness evaluation using Freesurfer | Assessing association between cortical thickness of "AD vulnerable" regions and cognitive reserve composite score | Cognitive reserve and cortical thickness were each independently associated with risk of clinical symptom onset within 7 years of baseline Interaction between cognitive reserve and mean cortical thickness for risk of progression more than 7 years from baseline |
| Pizzi et al., 2016 | 19 DLB, 15 AD, 19 HC | Subcortical structures and hippocampus segmentation and cortical thickness evaluation using Freesurfer | Differences between groups in total hippocampal volume, hippocampal subfields volume and extrahippocampal structures thickness, correlations with clinical outcomes | Cornu ammonis and subiculum bilaterally damaged in AD and preserved in DLB Perirhinal cortex and parahippocampus damaged in DLB, but not in AD |
| Poulakis et al., 2018 | 299 AD | Cluster analysis using 148 cortical and 14 subcortical Freesurfer segmentations | Five AD atrophy subtypes identification and description | 2 typical subtypes: Minimal atrophy, Limbic predominant, Hippocampal sparing, 3 atypical subtypes: Diffuse 1 and Diffuse 2 Hippocampal sparing and typical AD subtypes - worse clinical progression in visuospatial, memory and executive cognitive functions |
| Racine et al., 2018 | 149 MCI (discovery sample, 167 MCI (validation sample) | Cortical thickness estimation in 9 AD cortical signature ROIs | Personalized AD cortical thickness index calculation (pADi), cut-point identification, pADi+ and pADi- group description | 83% of pADi+ in the validation sample were AD CSF biomarker positive and pADi+ (63%) were more likely to progress to AD dementia after 1 and 3 years |
| Risacher et al., 2017 | 229 AD | Cortical and subcortical structrures volumes extraction using Freesurfer, hippocampal volume to cortical volume ratio calculation | Three AD subtypes identification: hippocampal sparing, typical AD, limbic predominant; HV:CTV and its relationship to clinical variables assessment | Hippocampal sparing subtype: more AD-like hypometabolism on FDG-PET and poorer baseline executive function, faster subsequent clinical decline in certain scores Larger HV:CTV ratio associated with poorer baseline executive function and a faster slope of decline in certain scores (driven rather by cortical atrophy |
| Tijms et al., 2018 | 160 abnormal amyloid MCI, 62 abnormal amyloid HC with subjective cognitive decline | GM network analysis, Cox proportional hazard models | Comparison between subjects who remained stable and those who showed clinical progression | After a median time of 2.2 years, 122 subjects showed clinical progression Lower network parameter values were associated with increased risk for progression, with the strongest hazard ratio of 0.29 for clustering |
| Toniolo et al., 2018 | 53 AD, 62 aMCI, 34 HC | Cerebellum GM VBM using SUIT and SPM | Differences between groups in regional cerebellar GM volumes | Progression of cerebellar GM volume changes throughout a continuous spectrum from early to late clinical stages of AD Vermis and paravermian areas of the anterior and posterior lobes are involved since aMCI stage with a later involvement of the hemispheric part of the posterior lobes and Crus I in AD dementia patients only |
| Vuksanovic et al., 2018 | 213 AD, 202 HC | Cortical thickness and surface area in 68 cortical regions evaluated using Freesurfer | Network analysis using cortical thickness and surface area measures and comparison between groups | The changes from normal are global in character and are not restricted to fronto-temporal and temporo-parietal lobes in AD Coordinated increases in CT and SA that may compensate for corresponding impairment in functionally linked nodes The differences were more pronounced in the surface area network in AD |
| Wang et al., 2018 | 293 mutation carriers, 188 mutation noncarriers | Cortical and subcortical structures segmentation using Freesurfer | Cognition rate of change prediction | Baseline MRI hippocampus volume and its rate of change significantly associated with the change in cognition in mutation carriers |
| Weston et al., 2016 | 20 symptomatic FAD, 23 pre-symptomatic FAD, 42 HC | Cortical thickness evaluation using longitudinal Freesurfer pipeline | Identification of cortical thinning signature from symptomatic FAD, prediction of thinning rate and evaluation of differences in cortical thickness between mutation carriers and HC | Cortical signature: entorhinal cortex, inferior parietal cortex, precuneus, superior parietal cortex, superior frontal cortex and supramarginal gyrus Significant differences in mean cortical signature thickness between mutation carriers and HC 3 years before predicted symptom onset The earliest significant difference detectable 4 years preonset in the precuneus Baseline mean signature thickness predicted rate of subsequent thinning and correlated with presymptomatic cognitive change |
| Whitwell et al., 2019 | 42 atypical AD | Volumes calculation of selected ROIs | Finding associations between age and region volumes | Younger age associated with smaller parietal and lateral temporal volumes |
| Yi et al., 2016 | 391 AD, 201 MCI (35 progressive MCI, 160 stable MCI), 181 HC | Subcortical structures segmentation using FSL | Comparison between groups, progression to AD hazard ratio calculation | AD vs HC: all structures except the globus pallidus smaller MCI vs HC: amygdala, thalamus, putamen, nucleus accumbens and hippocampus smaller All subcortical structures except the globus pallidus showed a positive correlation with cognitive function (MMSE) Smaller volumes of the hippocampus and nucleus accumbens were associated with increased risk of progression from MCI to AD |
| Zhao et al., 2019 | 41 AD, 43 aMCI, 35 SCD, 42 HC | Whole hippocampus and hippocampal subfields segmentation using Freesurfer | Differences between groups, correlations with cognitive scores | CA1, subiculum, presubiculum, molecular layer and fimbria showed the trend toward sigificant volume reduction among four groups with the progression of AD Volume of left subiculum correlated with performance across AVLT measures |

*red: cross-sectional studies, green: longitudinal studies*
